# Nonlinear Reconstruction of Images from Patterns Generated by Deterministic or Random Optical Masks—Concepts and Review of Research

**DOI:** 10.3390/jimaging8060174

**Published:** 2022-06-20

**Authors:** Daniel Smith, Shivasubramanian Gopinath, Francis Gracy Arockiaraj, Andra Naresh Kumar Reddy, Vinoth Balasubramani, Ravi Kumar, Nitin Dubey, Soon Hock Ng, Tomas Katkus, Shakina Jothi Selva, Dhanalakshmi Renganathan, Manueldoss Beaula Ruby Kamalam, Aravind Simon John Francis Rajeswary, Srinivasan Navaneethakrishnan, Stephen Rajkumar Inbanathan, Sandhra-Mirella Valdma, Periyasamy Angamuthu Praveen, Jayavel Amudhavel, Manoj Kumar, Rashid A. Ganeev, Pierre J. Magistretti, Christian Depeursinge, Saulius Juodkazis, Joseph Rosen, Vijayakumar Anand

**Affiliations:** 1 Optical Sciences Center and ARC Training Centre in Surface Engineering for Advanced Materials (SEAM), School of Science, Computing and Engineering Technologies, Optical Sciences Center, Swinburne University of Technology, Hawthorn, Melbourne, VIC 3122, Australia; danielsmith@swin.edu.au (D.S.); soonhockng@swin.edu.au (S.H.N.); tkatkus@swin.edu.au (T.K.); sjuodkazis@swin.edu.au (S.J.); 2 PG & Research Department of Physics, Thiagarajar College, Madurai 625009, India; gopishiva62@gmail.com (S.G.); dhanalakshmi_physics@tcarts.in (D.R.); srinivasan.n_physics@tcarts.in (S.N.); 3 PG & Research Department of Physics, The American College, Madurai 625009, India; francisgracy646@gmail.com (F.G.A.); shakinajselva@gmail.com (S.J.S.); bealthambu10@gmail.com (M.B.R.K.); ssrinbanathan@gmail.com (S.R.I.); 4 Hee Photonic Labs, LV-1002 Riga, Latvia; naarereddy@gmail.com; 5 Laboratory of Nonlinear Optics, University of Latvia, Jelgavas 3, LV-1004 Riga, Latvia; rashid.ganeev@lu.lv; 6 Division of Biological and Environmental Sciences and Engineering, King Abdullah University of Science and Technology, Thuwal 23955-6900, Saudi Arabia; vinoth.balasubramani@kaust.edu.sa (V.B.); pierre.magistretti@kaust.edu.sa (P.J.M.); christian.depeursinge@kaust.edu.sa (C.D.); 7 School of Electrical and Computer Engineering, Ben-Gurion University of the Negev, Beer-Sheva 8410501, Israel; rsykumar5@gmail.com (R.K.); ni3dubey@gmail.com (N.D.); rosenj@bgu.ac.il (J.R.); 8 Institute of Physics, University of Tartu, W. Ostwaldi 1, 50411 Tartu, Estonia; aravind.simon.john.francis.rajeswari@ut.ee (A.S.J.F.R.); sandhra-mirella.valdma@ut.ee (S.-M.V.); praveen@iisertirupati.ac.in (P.A.P.); info.amudhavel@gmail.com (J.A.); manojklakra@gmail.com (M.K.); 9 Organic Optoelectronics Research Laboratory, Department of Physics, Indian Institute of Science Education and Research (IISER), Tirupati 517507, India; 10 School of Computing Science and Engineering, VIT Bhopal University, Bhopal 466114, India; 11 Tashkent Institute of Irrigation and Agricultural Mechanization Engineers, National Research University, Kori Niyozov Str. 39, Tashkent 100000, Uzbekistan; 12 Tokyo Tech World Research Hub Initiative (WRHI), School of Materials and Chemical Technology, Tokyo Institute of Technology, 2-12-1, Ookayama, Meguro-ku, Tokyo 152-8550, Japan

**Keywords:** holography, computational imaging, nonlinear reconstruction, Fresnel incoherent correlation holography, coded aperture imaging, rotating point spread function, diffractive optics, scattering

## Abstract

Indirect-imaging methods involve at least two steps, namely optical recording and computational reconstruction. The optical-recording process uses an optical modulator that transforms the light from the object into a typical intensity distribution. This distribution is numerically processed to reconstruct the object’s image corresponding to different spatial and spectral dimensions. There have been numerous optical-modulation functions and reconstruction methods developed in the past few years for different applications. In most cases, a compatible pair of the optical-modulation function and reconstruction method gives optimal performance. A new reconstruction method, termed nonlinear reconstruction (NLR), was developed in 2017 to reconstruct the object image in the case of optical-scattering modulators. Over the years, it has been revealed that the NLR can reconstruct an object’s image modulated by an axicons, bifocal lenses and even exotic spiral diffractive elements, which generate deterministic optical fields. Apparently, NLR seems to be a universal reconstruction method for indirect imaging. In this review, the performance of NLR isinvestigated for many deterministic and stochastic optical fields. Simulation and experimental results for different cases are presented and discussed.

## 1. Introduction

Imaging can be broadly classified into direct and indirect imaging. Traditional lens-based imaging systems are direct imagers that perform simple geometric transformations such as scaling, rotation, translation, etc., of the object’s image onto the image sensor in a single step [1]. Indirect-imaging methods such as computational imaging and holography perform complicated optical transformations, where every object point is transformed in the first step into either a special intensity distribution or a hologram recorded by an image sensor [2]. In the next step, a computational method reconstructs the recorded information into an object image [2,3,4]. The optical transformation usually reorganizes the object’s image into depth-specific and/or wavelength-specific data, which is reconstructed into 3D and/or color images by a computational algorithm. This collective effort of computational and optical transformations expands the imaging capability to multiple dimensions beyond the limits of the direct imager [2]. In recent years, much attention has been given to the development of spatially incoherent imaging techniques in indirect imaging mode due to the multitude of useful properties they exhibit, such as high imaging resolution and low imaging noises [2,3,4,5,6].

Incoherent imaging techniques can be further classified into interference-based [2,5] and noninterferometric [6]. Many of the current interference-based incoherent imaging methods employ the Fresnel incoherent correlation holography (FINCH) architecture to record incoherent digital holograms [5,7,8,9,10]. An alternative method for recording incoherent digital holograms is scanning holography [4]. In FINCH, light from each object point is split into two differently modulated waves using either spatial multiplexing [7] or polarization multiplexing [10]. The resulting hologram obtained as an accumulation of the interference patterns between the entire wave pairs is reconstructed into an object image by numerical backpropagation. FINCH requires at least three camera shots to reconstruct the object’s image without the background and twin image noises. FINCH has the capability to break the Lagrange invariant condition and achieve enhanced resolution, but has a lower temporal resolution due to the requirement of multiple camera recordings.

**Figure 1:**
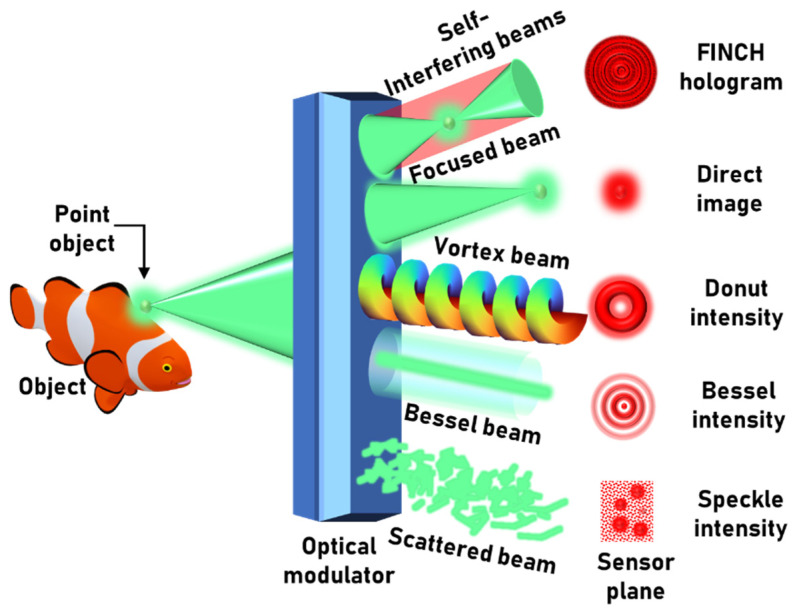
Optical configuration of imaging systems. The optical modulator can be a bifocal lens (FINCH), regular lens (direct imaging), spiral phase plate (vortex beam), an axicon (Bessel beam) or a random pinhole array (scattered beam).

The noninterferometric indirect-imaging methods include transport of intensity equations-based imaging [11,12], ghost imaging [13] and coded-aperture imaging (CAI) [14,15]. In this review, only CAI is considered. In CAI, the object intensity distribution is transformed into a specific intensity pattern using a coded mask followed by a computational reconstruction [14,15]. In the first reported studies by Ables [16] and Dicke [17] on coded apertures, people used a random array of pinholes to scatter light, generating artifacts and noise during the reconstruction. To understand the origin of these artifacts, it is necessary to understand the imaging process itself deeper. First, we assume that the imaging systems are linear shift-invariant systems and consist of three planes: object, optical modulator and sensor, as shown conceptually in Figure 1. The object intensity distribution generated for an object *O*, by the coded aperture, at the sensor plane can be expressed as *I_O_* = PSF⊗*O + N*, where ‘⊗’ is a 2D convolutional operator, PSF is the point spread function and *N* is a noise function. During deconvolution, the reconstructed image is given as $I_{R}={ℱ}^{-1}\left[ℱ\left(I_{O}\right)/ℱ\left(\mathrm{PSF}\right)\right]$, where ℱ and ${ℱ}^{-1}$ are the Fourier transform and inverse Fourier transform operators, respectively. Substituting for *I_O_* in the above equation, we obtain $I_{R}=O+{ℱ}^{-1}\left[ℱ\left(N\right)/ℱ\left(\mathrm{PSF}\right)\right]$. As seen from the last expression, the noise distribution in the reconstructed image might be amplified. In general, the Fourier transform of the scattered PSF might have values smaller than a noise distribution and usually has many nulls. This was the main drawback of CAI with a random array of pinholes.

Alternative correlation-based methods were developed to improve the reconstruction, in which the reconstructed image is given as $I_{R}=I_{O}*\mathrm{PSF}=\;\mathrm{PSF}*\mathrm{PSF}⊗O+N*\mathrm{PSF}$, where ‘∗’ is a 2D correlation operator. The above equation reduces to $I_{R}=\;\Lambda ⊗O+N*\mathrm{PSF}$, where Λ is a delta-like function that reconstructs the object image. The development of the Wiener filter improved the performance, but the noise was still a major concern [18]. The autocorrelation function was sharp but contained a background noise that set a limit on the signal-to-noise ratio. To avoid this problem, the Uniformly Redundant Array (URA) mask was proposed by Fenimore and Cannon, which yielded sharp autocorrelation functions with flat sidelobes [19]. Later, modified URA (MURA) with a double-exposure method was developed where the antimask was obtained by rotating the mask 180 degrees, aiding easy implementation of the two-shot-imaging method [20,21]. Later, CAI was adapted for spectral-imaging [22] and spatial-imaging applications [23].

Comparing CAI with FINCH, CAI is superior to FINCH in aspects of optical configuration, cost, size, number of optical components, speed and versatility. In a regular configuration, CAI cannot compete with FINCH’s resolution, but with some specially coded phase masks, the resolution of CAI can approach that of FINCH [24]. Even though both FINCH and CAI involved computational reconstruction, the computational transformation from complex FINCH hologram to 3D information is relatively more straightforward than that of CAI. FINCH and CAI evolved over many years, but the developmental areas were quite different. FINCH’s evolution was on developing novel architectures to improve SNR, lateral and axial resolution, reduction in path difference of interfering beams, and improvement of temporal resolution. The evolution of CAI was towards improving mainly the SNR, which was achieved using different types of masks and computational reconstruction mechanisms.

During the development of coded-aperture correlation holography (COACH) from FINCH [25] and its subsequent development into interferenceless-COACH (I-COACH) [26], a new computational reconstruction method, nonlinear reconstruction (NLR), was developed [27]. In NLR, the reconstructed image is given as

(1) $$I_{R}=\left|{ℱ}^{-1}\left\{{\left|\tilde{PSF}\right|}^{\alpha }\exp\left[j\cdot \arg\left(\tilde{PSF}\right)\right]{\left|{\tilde{I}}_{o}\right|}^{\beta }\exp\left[-j\cdot \arg\left({\tilde{I}}_{o}\right)\right]\right\}\right|,$$

where *α* and *β* are tuned to obtain the lowest entropy, arg(∙) refers to the phase and $\tilde{A}$ is the Fourier transform of *A*. The tuning of the magnitude of the two matrices improves the SNR beyond the limits of URA. This NLR was compared against different types of reconstruction methods such as matched filter (*α* = *β* = 1), phase-only filter (*α* = 0, *β* = 1), Wiener filter, regularized filter and maximum-likelihood algorithm [28,29]. In all the cases, NLR performed significantly better than the above algorithms. Surprisingly, NLR also enabled the conversion of FINCH from a three-shot technique to a single-shot one [30] and successfully reconstructed images when the light was modulated by axicons and spiral elements [31,32]. Furthermore, NLR also opened the possibility of reimplementing the coded aperture consisting of a random array of pinholes for imaging applications, but only this time with a high SNR [33,34,35].

In this review, FINCH holograms and intensity distributions of deterministic and random optical fields in the indirect-imaging framework are investigated using NLR. As it is known in spatially incoherent imaging systems, the phase information is redundant, and only self-interference can impact the imaging characteristics. For instance, a vortex beam with an azimuthal phase variation and a ring pattern with a uniform phase is expected to have the same effect in the proposed indirect-imaging framework. Different types of optical beams, such as Laguerre–Gaussian beams [36,37], Bessel beams [38], accelerated Airy beams [39], scattered beams [26] and self-interfering beams [40] are studied herein.

## 2. Methodology

Let us consider a point object emitting quasi-monochromatic light with an amplitude of $\sqrt{I_{o}}$ and located at a distance of *u* from the optical modulator at the point $\bar{r_{o}}=(x_{o},y_{o})$ on the object plane. The sensor plane is located at a distance of *v* from the optical modulator. The complex amplitude reaching the optical modulator is given as

(2) $$\psi_{1}=C_{1}\sqrt{I_{o}}Q\left(1/u\right)L\left(\bar{r_{o}}/u\right),$$

where

(3) $$\begin{array}{c} Q\left(1/u\right)=\exp\left[j\pi R^{2}/\left(\lambda u\right)\right],\\ L\left(\bar{o}/u\right)=\exp\left[j2\pi \left(o_{x}x+o_{y}y\right)/\left(\lambda u\right)\right], \end{array}$$

*C*_1_ is a complex constant and $R=\sqrt{x^{2}+y^{2}}$. The complex amplitude after the optical modulator is given as

(4) $$\psi_{2}=C_{1}\sqrt{I_{o}}L\left(\bar{r_{o}}/u\right)Q\left(1/u\right)\exp\left(-j\Phi_{\mathrm{OM}}\right),$$

where $\Phi_{\mathrm{OM}}$ is the phase of the optical modulator, which in the case of direct imaging is $\left(\pi R^{2}/\lambda \right)\left(1/u+1/v\;\right)$, creating an image of the object on the sensor plane [41]. For Bessel beam generation, the optical modulator is an axicon with a phase of $\exp\left[-j\left(2\pi \gamma R/\lambda \right)\left(n_{t}-1\right)\right]$, where *γ* is the base angle of the axicon and *n_t_* is the refractive index [42]. For vortex-beam generation, the optical modulator is a spiral Fresnel lens with a phase of $\exp\left[-j\left\{L\theta +\left(\pi R^{2}/\lambda \right)\left(1/u+1/v\;\right)\right\}\right]$, where *L* is the topological charge and θ is the azimuthal angle given as $\theta =\tan^{-1}\left(y/x\right)$ [43,44]. For the generation of a scattered beam, the optical modulator is a quasi-random lens with a phase given as $\exp\left[-j\left\{\left(\pi R^{2}/\lambda \right)\left(1/u+1/v\;\right)+\Phi_{R}\right\}\right]$, where *Φ_R_* is the random phase matrix with a particular scattering degree synthesized using Gerchberg–Saxton algorithm (GSA) [2,15,26]. For the generation of accelerating Airy beams, the optical modulator is a cubic phase mask with a phase given as $\exp\left[-j\left(2\pi /\lambda \right)\zeta \left(x^{3}+y^{3}\right)\right]$ [45]. For the generation of the FINCH hologram, the optical modulator has a phase function given as $M\exp\left[-j\left(\pi R^{2}/\lambda \right)\left(1/u+2/v\;\right)\right]+\left(1-M\right)\exp\left[-j\left(\pi R^{2}/\lambda \right)\left(1/u\;\right)\right]$, where *M* is a binary {0,1} quasi-random matrix and so (1 − *M*) is its antimask, which is mutually exclusive to *M*. The intensity pattern observed at a distance of *v* from the modulator is given as the magnitude square of a convolution of the complex amplitude beyond the modulator with the quadratic-phase function *Q*(1/*v*),

(5) $$I_{PSF}={\left|C_{2}\sqrt{I_{o}}L\left(\frac{\bar{r_{o}}}{u}\right)Q\left(\frac{1}{u}\right)\exp\left(-j\Phi_{\mathrm{OM}}\right)⊗Q\left(\frac{1}{v}\right)\right|}^{2},$$

where *C*_2_ is a complex constant. The sensor intensity for a 2D object *O* can be expressed as *I_O_* = *O*⊗*I_PSF_*. Unlike a coherent source, where the complex amplitude is convolved with PSF, here only the intensity distribution is convolved. Therefore, the object intensity pattern *I_O_* is formed by the replacement of every object point by *I_PSF_* followed by their summation. Consequently, there is no role for the phase profiles of the optical beams in this indirect imaging framework, and only the intensity distribution is considered. For example, a ring pattern generated by a lens-axicon pair [46,47] and a higher-order Laguerre–Gaussian beam will have the same imaging characteristics. The image reconstruction is carried out using NLR and optimized using the values of *α* and *β*. The imaging resolution in direct imaging mode is the diffraction-limited spot size ~1.22*λf*/*D*. The speckles formed by scattering have an average size of the diffraction-limited spot size. During autocorrelation, a peak with a width of twice the diffraction-limited spot is generated, which is equal to the diffraction-limited spot size when NLR was applied [27]. Therefore, there are two resolutions, namely optical and computational, and the computational resolution of NLR is usually higher than other computational reconstruction methods. The performance of the NLR in the case of various optical fields is studied in the following.

## 3. Simulation Results

**Figure 2:**
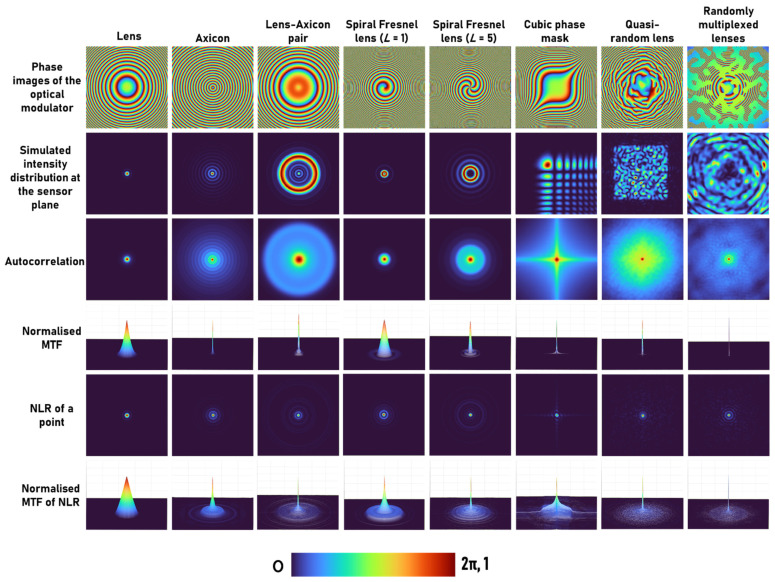
Comparison between different phase modulators according to functions and distributions that related to image reconstruction and resolution.

The simulation has been carried out in the far field with the following conditions: Matrix size of 500 × 500 pixels, λ = 0.65 μm, pixel pitch of 10 μm, *u* = ∞ and *v* = 50 cm. In this configuration, only 2D imaging is considered, and PSF is recorded by illuminating with collimated light on the diffractive element. The phase masks are designed for a diffractive lens with a focal length *f* = 50 cm, axicon with Λ = 150 μm, axicon-diffractive lens pair with an axicon period Λ = 800 μm, spiral Fresnel lens with topological charges *L* = 1 and *L* = 5 and with a focal length *f* = 50 cm, cubic phase mask with ζ = 491.3, quasi-random phase masks with a scattering ratio of σ = 0.1 and σ = 0.2 and randomly multiplexed bifocal lenses with focal lengths of 20 m and 25 cm, respectively, as is shown in Row 1 of Figure 2.

The Fresnel diffraction patterns were simulated for all the above cases. Autocorrelation and MTF are standards for describing the imaging characteristics of indirect imaging methods. Autocorrelation and MTF are related by a Fourier transform, and so a sharper autocorrelation generates a wider MTF and vice versa. In this study, the autocorrelation is compared with NLR, and the MTFs for both cases are investigated. The comparative results of the simulations with different apertures are shown in the various rows of Figure 2, according to the following list. Row 1: phase images of the various phase masks tested in the simulation. Row 2: The far-field diffraction patterns corresponding to the different phase masks. Row 3: The autocorrelation function |I_PSF_*I_PSF_|. The width of the autocorrelation function is approximately the lateral resolution of the indirect imaging system. Recalling the expression for reconstruction, $I_{R}={\;I}_{\mathrm{PSF}}{\;*\;I}_{\mathrm{PSF}}⊗O+N$, the autocorrelation function is the fundamental building block of the reconstructed image. Row 4: The modulation transfer function (MTF), which in direct imaging is $\mathrm{MTF}=c\left|ℱ\left(I_{\mathrm{PSF}}\right)\right|$, and in indirect imaging framework is $\mathrm{MTF}=c^{\prime }\left|ℱ\left(I_{\mathrm{PSF}}*I_{\mathrm{PSF}}\right)\right|$, where *c* and *c*’ are constants that guarantee the MTFs are normalized. Row 5: The NLR of a single point. Row 6: The MTF of the systems with NLR. Although the NLR violates the linearity of the imaging system, we define the MTF of such a system as the normalized magnitude of the Fourier transform of the point image. The reconstructed image due to the NLR is

(6) $$I_{R}={ℱ}^{-1}\left\{{\left|{\tilde{I}}_{PSF}\right|}^{\alpha }\exp\left[j\cdot \arg\left({\tilde{I}}_{PSF}\right)\right]{\left|{\tilde{I}}_{o}\right|}^{\beta }\exp\left[-j\cdot \arg\left({\tilde{I}}_{o}\right)\right]\right\}$$

For an object of a point *I_o_* = *I_PSF_*, and recall that ${\tilde{I}}_{PSF}=H*H,$ where $H=\exp\left(-j\Phi_{\mathrm{OM}}\right)$ is the transfer function of the modulator, the MTF of the systems with NLR is $\mathrm{MTF}={\left|H*H\right|}^{\alpha +\beta }$. Comparing the various rows of Figure 2, it is clear that the NLR of a point is sharper than the conventional autocorrelation function and the MTF of the NLR is wider than the conventional MTF in all the cases of different modulators. According to these observations, it is expected that the image resolution of the NLR is superior to the conventional techniques, although the numerical aperture is identical for the entire optical modulators and techniques. Note that in all previous studies, the image resolution of NLR was found to be higher than the other tested methods [28,29].

**Figure 3:**
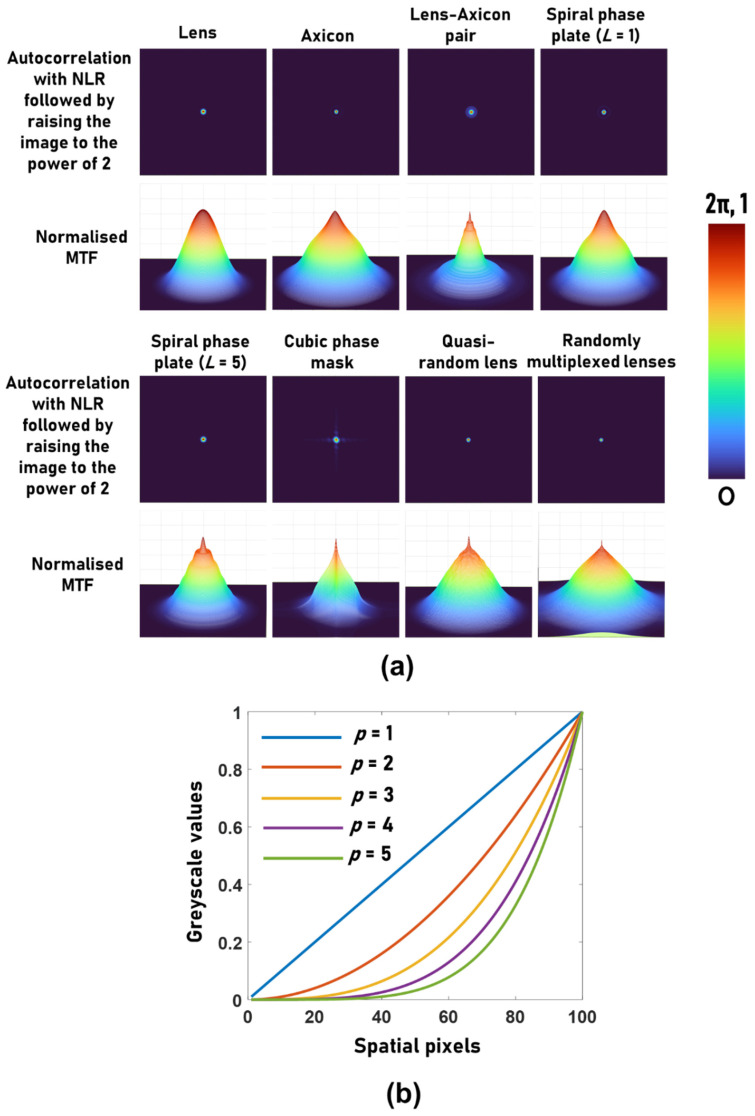
(**a**) Autocorrelation with NLR followed by raising the image to the power of *p* = 2 and their respective MTF profiles. (**b**) The influence of *p* on a grayscale slope.

Another important observation is that the peak-to-background ratio (PBR) is significantly higher in NLR when compared to the reconstruction with the matched filter (*α* = *β* = 1), as shown in Table 1. This high PBR makes the method suitable for the reconstruction of high-contrast objects or objects with binary values by the application of an additional operation *I_R,p_* = (*I_R_*)*^p^*, which suppresses the background information. In the Fourier domain, the above operation can be expressed as a convolution resulting in an increase in the bandwidth. For *p* = 2, if *G* is the MTF corresponding to *I_R_*, then G*_p_*$=ℱ\left[{ℱ}^{-1}\left(G\right)\times {ℱ}^{-1}\left(G\right)\right]=G⊗G$. Therefore, with each increase in *p*, the bandwidth increases by the bandwidth of *G*. The influence of this process on imaging is examined in the following. This process is suitable only for objects with binary values and is detrimental for objects with greyscale values. In fact, most, if not all, of the previous applications of NLR to stochastic—as well as deterministic—optical fields involved only binary objects such as standard-resolution targets [2,27,30,31,32,34]. The reconstructed point and normalized MTF after the application of the above method for *p* = 2 with NLR for eight cases of Figure 2 are shown in Figure 3a. As seen from the results, raising the image to the power of 2 improves the MTF. The variation in the greyscale values when *p* was varied from 1 to 5 is shown in Figure 3b. As seen in Figure 3b, with an increase in the value of *p*, the greyscale profile changes from linear to nonlinear.

**Figure 4:**
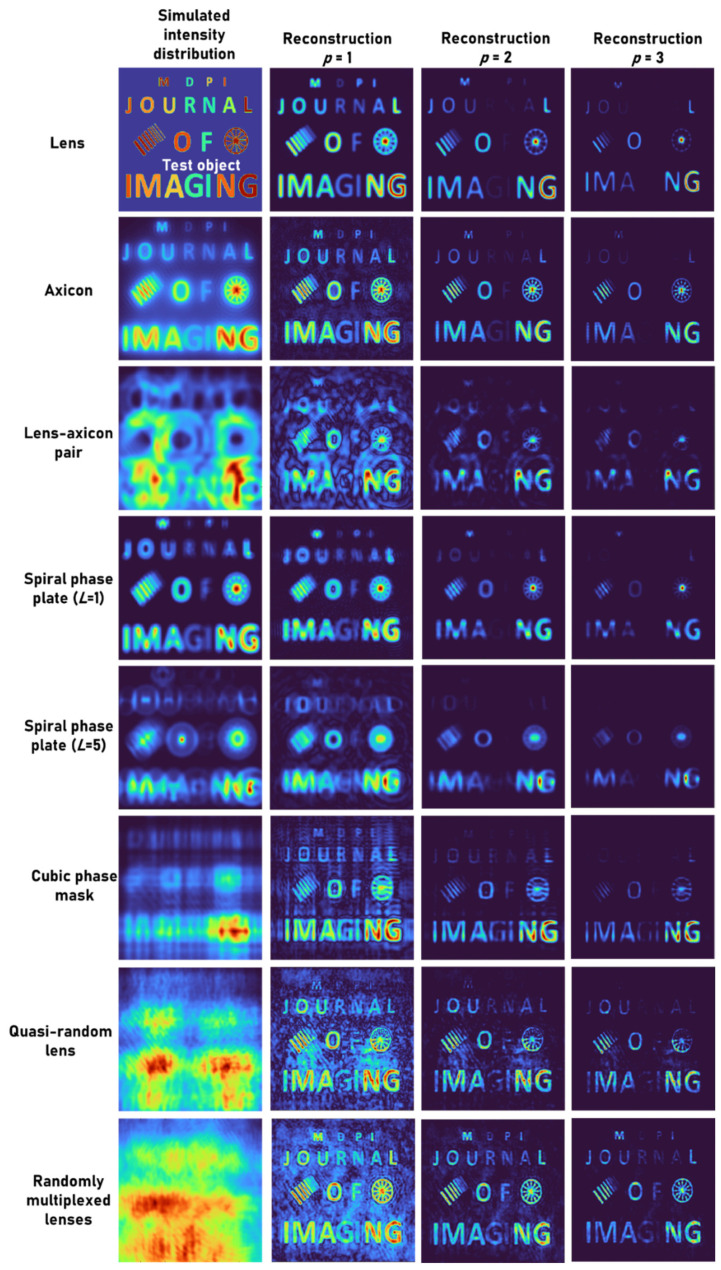
Simulated intensity distribution for a test object and the reconstruction results for *p* = 1, 2 and 3.

A test object, “MDPI JOURNAL OF IMAGING”, with varying font sizes and different gray levels, was used. Two objects: grating with varying periods and a wheel-like object, were added to the input picture. The intensity distribution simulated for different cases and the reconstruction results for *p* = 1, 2 and 3 are shown in Figure 4. The mask characteristics of different beams are expressed in the respective intensity distributions. As is seen in Figure 4, when the *p*-value increased, the PBR and visibility improved while the grey level profile varied. Once again, this proves that the application of raising the image to the power of *p* is suitable for binary objects, but with grayscale objects, the contrast of the reconstructed images is varied.

The simulation results show that as small-size elements approach the resolution limit, the image intensity decreases, and raising the image to the power of p suppresses small elements in the reconstructed picture. Comparing the outcomes of different cases also reveals several interesting properties. When observing the spokes of the wheel, imaging using an axicon enhances such fine features, which are suppressed in the case of a diffractive lens. The performance of the lens–axicon pair and the spiral Fresnel lens for *L* = 5 with NLR is lower in comparison to the other cases. The cubic phase mask resolved the grating lines better than in the other cases. The randomly multiplexed lenses retained not only the grayscale information but also exhibited a high computational resolution. The overall observation reveals that even though the same information is transferred into the aperture, in the indirect imaging framework with NLR, different optical fields performed differently.

## 4. Experimental Results

Experiments on the indirect-imaging framework with stochastic and deterministic optical fields have been carried out by different authors of this article. Some of the demonstrations involved a spatial light modulator (SLM), while in others, people used diffractive elements fabricated using different methods, ranging from femtosecond ablation and electron beam lithography to lens grinding.

The entire electro-optical experiments for each mask include recording the PSF, but the imaging of the target is performed by a digital convolution between the target matrix and the experimental PSF. Some of the PSFs are recorded when a pinhole is illuminated by coherent laser light, and others are obtained under incoherent light. However, all the digital processes of recording the object response are performed under the rules of incoherent imaging as convolutions between intensity functions.

### 4.1. Lensless I-COACH

**Figure 5:**
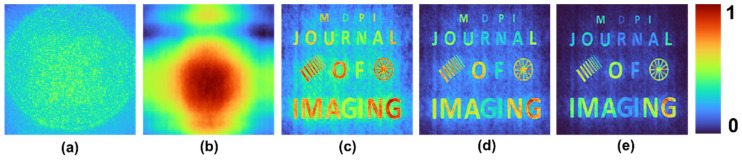
(**a**) Recorded PSF and (**b**) object intensity response. Reconstruction results using (**c**) NLR (*p* = 1), (**d**) NLR (*p* = 2) and (**e**) NLR (*p* = 3) for LI-COACH with a QRL.

In the line of development of I-COACH [26], after COACH [25], Lensless I-COACH (LI-COACH) [48] was developed. In the proposed LI-COACH, the only optical component between the object and the sensor is a quasi-random lens (QRL) mounted with a spacing of 26 cm between adjacent components. The QRL was designed using a modified GSA, where the Fourier transform was replaced by the Fresnel transform [49,50]. The setup of LI-COACH consisted of an optical channel illuminated by light-emitting diodes (LED) (Thorlabs LED631E, 4 mW, λ = 635 nm, Δλ = 10 nm). In the first step, the PSF was recorded using a pinhole (*φ* = 100 μm). The QRL was displayed on an SLM (Holoeye PLUTO, 1920 × 1080 pixels, 8 μm pixel pitch, phase-only modulation), and the light from the pinhole was polarized along the active axis of the SLM. The intensity distribution was captured by an image sensor [pco.edge 5.5 scientific CMOS (sCMOS), 2560 × 2160 pixels, 6.5 μm pixel pitch]. The intensity patterns of the PSF and the object’s response are shown in Figure 5a and 5b, respectively. The reconstruction results using NLR (α = 0.2, β = 1) for *p* = 1, 2 and 3 are shown in Figure 5c–5e, respectively. As expected, the visibility improved, and a slight variation in the greyscale profile was observed.

### 4.2. Random Array of Pinholes

**Figure 6:**
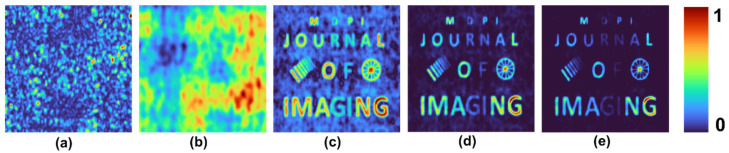
(**a**) Recorded PSF, (**b**) object’s response pattern. Reconstruction results using (**c**) NLR (*p* = 1), (**d**) NLR (*p* = 2) and (**e**) NLR (*p* = 3) for random array of pinholes.

A mask containing a random array of 2000 pinholes, each with an average diameter of 80 μm, was fabricated using Intelligent Micropatterning SF100 XPRESS on a chromium-coated glass plate. The diameter of the mask pattern was about 8 mm. A LED source (M617L3, λ_c_ = 617 nm, FWHM = 18 nm) was used for illumination. The PSF (pinhole φ = 100 μm) was recorded when the distances between the object and the mask containing a random array of pinholes and between the mask and the sensor plane (DCU223M, 1024 × 768 pixels, pixel size = 4.65 μm) were both 10 cm. The intensity patterns of the PSF, the object’s response and reconstruction results of NLR (α = 0, β = 0.6) for *p* = 1, 2 and 3 are shown in Figure 6a–e, respectively.

### 4.3. QRL Fabricated Using Electron-Beam Lithography

**Figure 7:**
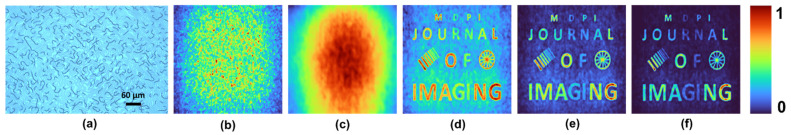
(**a**) Optical microscope image of the central part of the QRL fabricated using electron-beam lithography. (**b**) Recorded PSF, (**c**) object’s response pattern. Reconstruction results using (**d**) NLR (*p* = 1), (**e**) NLR (*p* = 2) and (**f**) NLR (*p* = 3).

A QRL was fabricated using electron-beam lithography (RAITH 150^TWO^) with a diameter of 5 mm and focal length of 5 cm with a binary-phase profile [51], as shown in Figure 7a. The same LED source as in the previous section was used for illumination. The distance between the pinhole (*φ* = 100 μm) and the QRL was 10 cm. The image sensor was located at a distance of 10 cm from the QRL. The intensity patterns of the PSF, the object’s response and the reconstruction results of NLR (*α* = 0, *β* = 0.6) for *p* = 1, 2 and 3 are shown in Figure 7b–f, respectively.

### 4.4. QRL Fabricated by Grinding Lens

**Figure 8:**
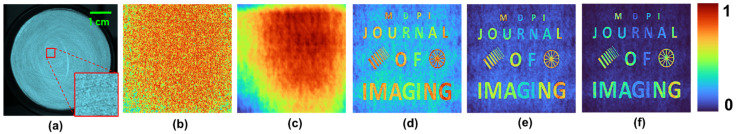
(**a**) Image of the QRL fabricated using lens grinding with sandpaper. (**b**) Recorded PSF, (**c**) object’s response pattern. Reconstruction results using (**d**) NLR (*p* = 1), (**e**) NLR (*p* = 2) and (**f**) NLR (*p* = 3).

A QRL was fabricated using lens grinding. A refractive lens with a focal length of 10 cm was ground using sandpaper with different grit sizes. The grinding was carried out manually in all directions to achieve a uniform scattering. The image of the top surface of the QRL with a minimum feature size of 100 μm is shown in Figure 8a. In this case, a laser source emitting at 632 nm was used. The recorded scattered intensity distribution at the focal plane of the lens is shown in Figure 8b. The intensity patterns of the object’s response and the reconstruction results of NLR (*α* = 0, *β* = 0.6) for *p* = 1, 2 and 3 are shown in Figure 8c–f, respectively.

Comparing all the above cases in Section 4.1, Section 4.2, Section 4.3 and Section 4.4, there was only a slight variation in background noise, with no peculiar behavior observed. The improvement in PBR and visibility was observed with an increase in *p* with a slight variation in greyscale profile, as expected.

### 4.5. Photon-Sieve Axicon

**Figure 9:**
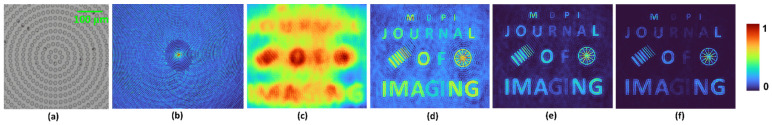
(**a**) Image of the photon-sieve axicon fabricated using femtosecond ablation. (**b**) Recorded PSF, (**c**) object’s response pattern. Reconstruction results using (**d**) NLR (*p* = 1), (**e**) NLR (*p* = 2) and (**f**) NLR (*p* = 3).

A photon-sieve axicon is a binary axicon where the rings are composed of discs [31]. A photon-sieve axicon with a period of ~20 μm and diameter of 5 mm was fabricated using femtosecond ablation on a sapphire substrate with a thickness of 500 μm. The optical microscope image of the central part of the fabricated device is shown in Figure 9a. The recorded scattered intensity distribution at 5 mm from the axicon is shown in Figure 9b. The intensity patterns of the object’s response and the reconstruction results of NLR (α = 0, β = 0.6) for *p* = 1, 2 and 3 are shown in Figure 9c–f, respectively.

### 4.6. Diffractive Lens

**Figure 10:**
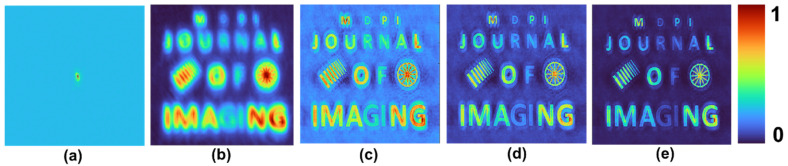
(**a**) Recorded PSF, and (**b**) object’s response pattern. Reconstruction results using (**c**) NLR (*p* = 1), (**d**) NLR (*p* = 2) and (**e**) NLR (*p* = 3) for a diffractive lens.

A diffractive lens with a focal length of 50 cm was displayed on an SLM (Holoeye-PLUTO-2.1, Phase-only spatial light modulator 1920 × 1080 pixel, Δ = 8 μm) and illuminated by a spatially filtered and a collimated laser beam [THORLABS, LDM635 laser diode with λ = 635 nm, Power = 4.0 mW, beam size at the source end (elliptical) 3 mm × 5 mm], and recorded by a sensor (Spiricon SP-928 beam profiling camera, 1928 × 1448, Δ = 3.69 um) with a slight focal point aberration. The out-of-focus point image is shown in Figure 10a. The out-of-focus image of the test object and the reconstruction results obtained by NLR (*α* = 0, *β* = 0.6) for *p* = 1, 2 and 3 are shown in Figure 10b–e, respectively.

### 4.7. Spiral Fresnel Lens

**Figure 11:**
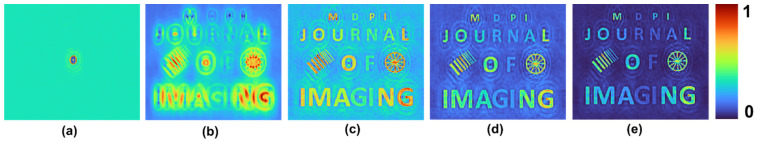
(**a**) Recorded PSF, (**b**) object’s response pattern. Reconstruction results using (**c**) NLR (*p* = 1), (**d**) NLR (*p* = 2) and (**e**) NLR (*p* = 3) for a spiral Fresnel zone lens with *L* = 1.

**Figure 12:**
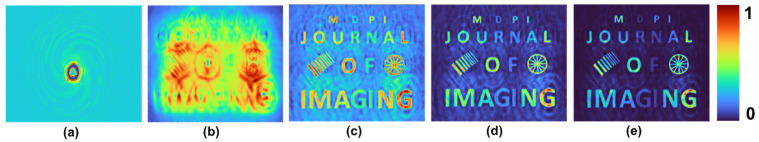
(**a**) Recorded PSF, (**b**) object’s response pattern. Reconstruction results using (**c**) NLR (*p* = 1), (**d**) NLR (*p* = 2) and (**e**) NLR (*p* = 3) for a spiral Fresnel zone lens with *L* = 5.

Spiral Fresnel lenses with *L* = 1 and 5 and a focal length of 50 cm were displayed on an SLM and illuminated by a laser similar to Section 4.1. The intensity pattern recorded for *L* = 1 by the sensor is shown in Figure 11a. The object’s intensity at 50 cm from the SLM and the reconstruction results by NLR (*α* = 0, *β* = 0.6) for *p* = 1, 2 and 3 are shown in Figure 11b–e, respectively. The target’s image is edge-enhanced, as expected. The intensity pattern recorded for *L* = 5 by the sensor is shown in Figure 12a. The target’s intensity distribution is shown in Figure 12b. The reconstruction results for NLR (*α* = 0, *β* = 0.6) for *p* = 1, 2 and 3 are shown in Figure 12c–e, respectively.

### 4.8. Lens–Axicon Pair

**Figure 13:**
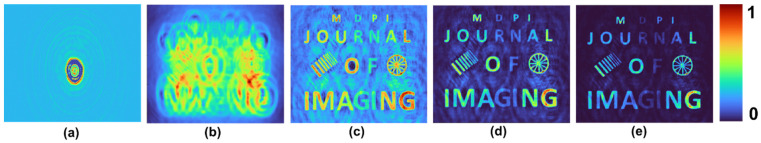
(**a**) Recorded PSF, (**b**) object’s response pattern. Reconstruction results using (**c**) NLR (*p* = 1), (**d**) NLR (*p* = 2) and (**e**) NLR (*p* = 3) for lens–axicon pair.

A lens–axicon pair with a focal length of 50 cm and an axicon period of ~320 μm was displayed on the SLM, similar to Section 4.7. The recorded PSF, object’s response at 50 cm from the SLM and reconstructed results of NLR (α = 0, β = 0.6) for *p* = 1, 2 and 3 are shown in Figure 13a–e, respectively.

### 4.9. FINCH with Polarization Multiplexing

**Figure 14:**
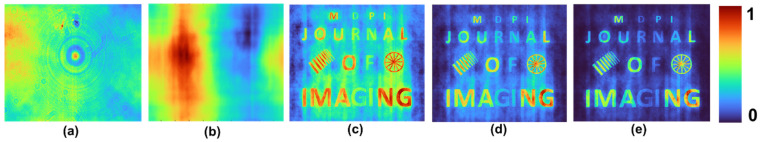
(**a**) Recorded PSF, (**b**) object’s response pattern. Reconstruction results using (**c**) NLR (*p* = 1), (**d**) NLR (*p* = 2) and (**e**) NLR (*p* = 3) for FINCH with random multiplexing configuration.

FINCH setup was built in polarization-multiplexing configuration [52]. The light from an object is polarized at 45° with respect to the active axis of the SLM, and a quadratic phase mask is displayed on the SLM. Therefore, at the SLM, two beams are generated: modulated and unmodulated beams, which are interfered at the sensor plane using a second polarizer oriented at 45° with respect to the active axis of the SLM [52]. The experimental setup uses a collimated LED emitting at 532 nm (FWHM = 35 nm) as an illumination source. An optical lens (focal length = 10 cm) is placed to critically illuminate the pinhole at the object plane. The light from the pinhole is polarized to 45° orientation by a polarizer and collimated. The beam is reflected by a phase-only SLM (Pixels: 1920 × 1080, Pixel pitch: 8 µm), which displays a diffractive lens with a focal length of 20 cm. A second polarizer is perpendicular to the first polarizer so that the modulated and the unmodulated beams can interfere with each other. An image sensor (1392 × 1040 pixels with 6.45 µm square pixels) is placed at the hologram plane at 40 cm from the SLM to capture the holograms digitally for the numerical reconstruction. The recorded PSF, object’s intensity response and reconstruction results of NLR (α = 0, β = 0.6) for *p* = 1, 2 and 3 are shown in Figure 14a–e, respectively.

### 4.10. FINCH with Spatial Random Multiplexing

**Figure 15:**
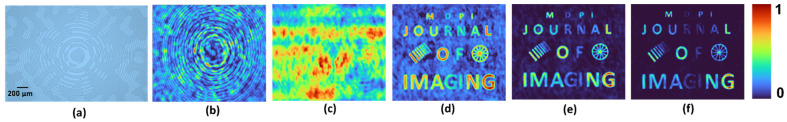
(**a**) Optical microscope image of the randomly multiplexed bifocal diffractive lenses. (**b**) Recorded PSF, (**c**) object’s response pattern. Reconstruction results using (**d**) NLR (*p* = 1), (**e**) NLR (*p* = 2) and (**f**) NLR (*p* = 3) for FINCH with spatial random multiplexing configuration.

A randomly multiplexed bifocal lens was designed and fabricated using electron-beam lithography (RAITH150^TWO^) with focal lengths of 5 cm and 10 cm and a diameter of 5 mm. A pinhole with a size of 20 μm was mounted at 5 cm from the diffractive element. Around 50% of the light was collected and focused at 5 cm from the diffractive element by the lens with a focal length of 10 cm, and the remaining was collimated. An image sensor (Thorlabs DCU223M, 1024 pixels × 768 pixels, pixel size = 4.65 μm) was used for recording the hologram at a distance of 10 cm from the diffractive element. The optical microscope image of the diffractive element is shown in Figure 15a. The PSF, object’s intensity response, and reconstruction results of NLR (α = 0, β = 0.6) for *p* = 1, 2 and 3 are shown in Figure 14b–f, respectively [30].

### 4.11. Double-Helix Beam with Rotating PSF

**Figure 16:**
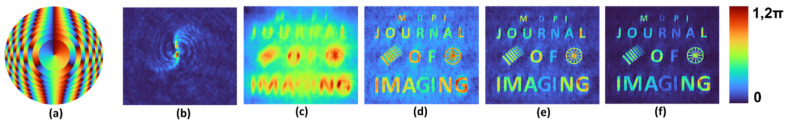
(**a**) Phase image of the multifunctional DOE. (**b**) Recorded PSF, (**c**) object’s response pattern. Reconstruction results using (**d**) NLR (*p* = 1), (**e**) NLR (*p* = 2) and (**f**) NLR (*p* = 3) for double-helix beam with rotating PSF.

A spiral element [53] with a phase distribution shown in Figure 16a was used as an optical modulator along with a diffractive lens. An LED source (Thorlabs LED625L, 12 mW, λ = 625 nm, ∆λ = 15 nm) was used for critically illuminating the pinhole. The SLM (Holoeye PLUTO, 1920 × 1080 pixels, 8 µm pixel pitch, phase-only modulation) was used to modulate the light beam by displaying the phase of the spiral element along with the lens function having a focal length of 14 cm. The distance between the SLM and the digital camera (Retiga R6-DCC3260M, pixel size 4.54 μm × 4.54 μm) was 14 cm. A polarizer was used to only allow light along the active axis of the SLM. The image of the recorded PSF, object’s response and reconstructed results of NLR (*α* = 0, *β* = 0.6) for *p* = 1, 2 and 3 are shown in Figure 16b–f, respectively [32].

## 5. Discussion and Conclusions

The optical fields that were simulated and experimentally generated in the previous sections are widely used for various applications such as optical trapping (Laguerre–Gaussian beams), 3D fabrication (Bessel beam), corneal surgery (Lens–axicon pair) and imaging through occlusion and turbid media (Bessel and accelerating Airy beams). Most, if not all, the light–matter interactions have been observed using another optical channel, which is the imaging channel. The proposed direction of research using the above deterministic optical beams in an indirect-imaging framework may compactify the future optical systems by avoiding the imaging channel. It is convincing from the simulation and previous experimental studies [2,27,28,30,31,32,33,34,35] that the NLR, and raising the image to a power of *p*, can reconstruct a high-contrast object’s image faithfully and at the same time act as a spectral and spatial confocal system. Raising the image to a power of *p* improved the visibility with a slight decrease in the greyscale profile. With the latest developments in deep-learning-based image enhancement, we believe that NLR, and raising the image to a power of *p* and deep-learning methods, can act as a universal reconstruction method for imaging in indirect-imaging framework in the future [54].

In this review, we have investigated several known optical apertures that have the potential for different applications in the indirect-imaging framework using NLR. Simulation and experimental results indicate that NLR is a universal reconstruction method when combined with raising the image to a power of *p* (*p* is an integer equal to or greater than 1). As shown in the simulation and experimental results, the PBR and visibility are improved with increasing *p*, while the greyscale profile varies. It is also noted that different beams enhance or suppress different details of the object image. This leads to an important question: Can information be transformed simultaneously into different types of beams leading to an overall improvement in the reconstruction? This type of hybridization has been investigated in the past, which resulted in creating FINCH-COACH states with nonlinear imaging characteristics [55]. We believe that other hybridizations might benefit better performance. The 3D performances have not been compared, which may be an interesting study in the future. While some of the known beams have been studied in this review, there are numerous scalar beams as well as vector beams developed with exotic characteristics, and it will be interesting to study such beams in the indirect-imaging framework. With the development of new materials-engineering and fabrication methods, this indirect-imaging framework and NLR can be extended to optical fields that have variations in polarization as well [56,57,58].

Comparing the results of NLR for different optical fields, it is seen that the performance was best for a direct-imaging system using a lens. Considering this fact and the broad applicability of NLR with slight variations, the review also proposes another important question: What is the optimal PSF for NLR? In addition to what has been discussed in this review, there are other optical modulators that generate a random array of spots [59], a random array of FINCH holograms [60], a ring pattern [61] and new reconstruction algorithms that are based on NLR [62], which are topics of future investigation. We believe that we have introduced the topic of indirect imaging using deterministic and stochastic optical fields extensively in detail, and have concluded with interesting questions that may lead to further research in this area.

## Figures and Tables

**Table 1:** Rounded PBR values obtained for different phase masks for autocorrelation and NLR.

Peak-to-Background Ratio	Lens	Axicon	Lens–Axicon Pair	Spiral Fresnel Zone Lens *L* = 1	Spiral Fresnel Zone Lens *L* = 5	Cubic Phase Mask	Quasi-random Lens	Randomly Multiplexed Lenses
Autocorrelation	2518	8	99	925	262	61	23	10
NLR	5957	5258	3472	5739	4068	18,147	7565	5977

## Data Availability

The data are contained within this article.
